# A comparison of stimulus types in online classification of the P300 speller using language models

**DOI:** 10.1371/journal.pone.0175382

**Published:** 2017-04-13

**Authors:** William Speier, Aniket Deshpande, Lucy Cui, Nand Chandravadia, Dustin Roberts, Nader Pouratian

**Affiliations:** 1 Department of Neurosurgery, University of California, Los Angeles, Los Angeles, CA, United States of America; 2 Department of Bioengineering, University of California, Los Angeles, Los Angeles, CA, United States of America; 3 Neuroscience Interdepartmental Program, University of California, Los Angeles, Los Angeles, CA, United States of America; 4 Brain Research Institute, University of California, Los Angeles, Los Angeles, CA, United States of America; Duke University, UNITED STATES

## Abstract

The P300 Speller is a common brain-computer interface communication system. There are many parallel lines of research underway to overcome the system’s low signal to noise ratio and thereby improve performance, including using famous face stimuli and integrating language information into the classifier. While both have been shown separately to provide significant improvements, the two methods have not yet been implemented together to demonstrate that the improvements are complimentary. The goal of this study is therefore twofold. First, we aim to compare the famous faces stimulus paradigm with an existing alternative stimulus paradigm currently used in commercial systems (i.e., character inversion). Second, we test these methods with language model integration to assess whether different optimization approaches can be combined to further improve BCI communication. In offline analysis using a previously published particle filter method, famous faces stimuli yielded superior results to both standard and inverting stimuli. In online trials using the particle filter method, all 10 subjects achieved a higher selection rate when using the famous faces flashing paradigm than when using inverting flashes. The improvements achieved by these methods are therefore complementary and a combination yields superior results to either method implemented individually when tested in healthy subjects.

## 1 Introduction

The P300 Speller is a common brain-computer interface (BCI) system that provides a means of communication for patients with high brain stem injuries or motor neuron diseases such as amyotrophic lateral sclerosis (ALS) [1]. The system relies on electroencephalogram (EEG) detection of evoked responses to rare target stimuli to identified intended letters for communication. Because the signal to noise ratio (SNR) is low, several trials must be combined in order to correctly classify responses. The resulting typing speed can therefore be slow, prompting many studies focused on system optimization. Approaches include varying the grid size [2–4], optimizing interstimulus interval (ISI) [5,6], and adopting different signal processing methods [7–10].

One active area of research has been to modify the type of visual stimulus used. In the original system, the character grid is gray and the intensified characters are changed to white. However, other types of visual stimuli could potentially elicit stronger P300 or other stimulus evoked responses and several studies have aimed to show superior flashing methods by using character motion [11], modifying character size and sharpness [11], changing stimulus colors [12], varying the grid layout [13], or increasing stimulus contrast [14]. The most successful stimulus to date has been the presentation of “famous faces” [15]. In this system, stimuli consist of overlaying characters with images of a famous face. This method is based on previous findings that face recognition has been found to elicit two evoked responses in addition to the P300: the N170 and N400f [16]. By incorporating face images, the response signals elicited are more salient, leading to a reduction in the number of stimuli required for perfect accuracy by over 45%, greatly improving typing speed [15]. While the improvement using “famous faces” was significant over the traditional system, to our knowledge it has not been compared to other alternative stimuli. Moreover, while it has been validated online [17], it was only using a traditional classifier and does not reflect the true performance of an online BCI system using state of the art classification methods.

Separately, recent work has involved the incorporation of language information into the signal classifier [18]. This movement in BCI research integrates knowledge about the domain of natural language to improve classification, similar to methods used in other domains such as speech recognition [19]. Several BCI studies have shown incremental improvements in system speed and accuracy using n-gram language models, first using naïve Bayes [20,21] and later using a partially observable Markov decision process [22] and a hidden Markov model [23,24]. Recently, a particle filter (PF) algorithm was introduced which allowed for the use of more complicated language models to further improve results [25]. This method approximates distributions by projecting samples through a state-space language model based on the observed EEG signals [26]. The system then determines the most likely output by finding the state that attracts the highest number of samples. In offline trials, this method yielded an increase in typing speed from 5.87 characters/minute to 8.70 characters/minute over a system without language model integration.

While both famous faces stimuli and language model integration have been shown separately to provide significant improvements, the two methods have not yet been implemented together to demonstrate that the improvements are complimentary. It is conceivable, for instance, that SNR could be improved to the point where perfect classification would be possible from the signal alone and adding a bias based on prior knowledge would not provide any benefit. It is necessary to test these methods together in order to verify that the combination is indeed better than the individual components.

The goal of this study is therefore twofold. First, we aim to compare the famous faces stimulus paradigm with an existing alternative stimulus paradigm currently used in commercial systems such as the Intendix speller (Guger Technologies, Graz, Austria). This comparison is necessary because, while the superiority of the famous faces paradigm over traditional stimuli has been previously established, it has not been compared to other paradigms that are in current use. Second, we will test these methods with language model integration to see if the advances reported in these two research areas can be combined to further improve BCI communication. We hypothesized that using famous face stimuli will increase the speed and accuracy of the P300 speller system over other stimulus paradigms and that incorporating both famous face stimuli and a language model classifier will combine to yield superior performance than either method individually.

## 2 Materials and methods

### 2.1 Data collection

All data was acquired using g.tec amplifiers, active EEG electrodes, and electrode cap (Guger Technologies, Graz, Austria); sampled at 256 Hz, referenced to the left ear; grounded to AF_Z_; and filtered using a band-pass of 0.1–60 Hz. The electrode set consisted of 32 channels placed according to a previously published configuration (Fpz, Fz, FC1, FCz, FC2, FC4, FC6, C4, C6, CP4, CP6, FC3, FC5, C3, C5, CP3, CP5, CP1, P1, Cz, CPz, Pz, POz, CP2, P2, PO7, PO3, O1, Oz, O2, PO4, PO8) [5]. The system used a 6 × 6 character grid, row and column flashes, and a stimulus onset asynchrony of 125 ms (consisting of a 100 ms flash duration and a 25 ms interstimulus interval). After each stimulus, the next 600 ms of data from each of the 32 channels were used as features for classification.

This research was approved by the University of California, Los Angeles institutional review board (IRB), IRB#11–002062. Written consent was obtained from all subjects using a consent form approved by the IRB. The subjects in this study consisted of 25 healthy volunteers with normal or corrected to normal vision between the ages of 20 and 35. Fifteen of the subjects participated in a preliminary study comparing the inverting and non-inverting paradigms and the remaining 10 used the inverting and famous faces paradigms. For each of the stimulus paradigms, the training sessions consisted of three sessions of copy spelling 10 character phrases each for the inverting and famous faces paradigms. The approaches were counterbalanced across subjects to account for possible order or fatigue effects. In the main experiment, each subject then chose a target phrase to spell in online sessions, during which the subject had five minutes to spell as much of the phrase as they could using both stimulus paradigms. Subjects were instructed not to correct errors and to repeat the phrase if they completed it in under five minutes. The training data was then analyzed retrospectively using three-fold cross-validation to provide an additional offline comparison of results using the two stimulus paradigms when using classifiers with and without a language model.

BCI2000 was used for data acquisition and online analysis [27]. Offline analysis was performed using MATLAB (version 8.6.0, MathWorks, Inc, Natick, MA).

### 2.2 Interface

**Fig 1:**
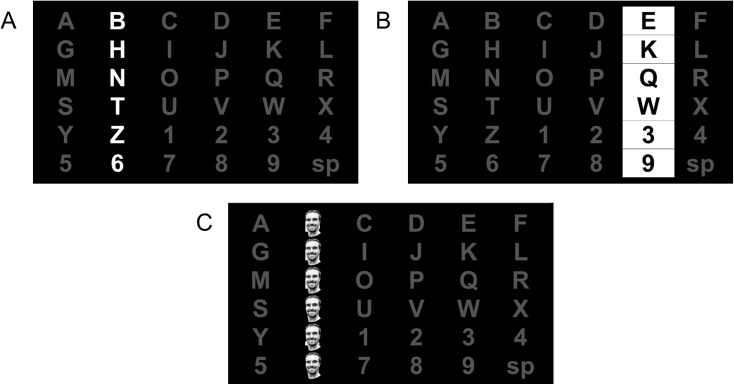
**Screenshots of a stimulus presentation using Non-Inverting (a), Inverting (b), and Famous Faces (c).** In the experiment, an image of Einstein was used for the famous faces paradigm, which is replaced here with an image of one of the authors due to print license. The individual pictured has given written informed consent (as outlined in the PLOS consent form) to publish their image.

Three stimulus types are compared in this study. The first method is the standard method, consisting of highlighting flashed characters by “intensifying” the font color to white (Fig 1A) [1]. The second method is letter inversion, or changing the background to white and the character to black (Fig 1B). The third method overlays the character with an image of a face as proposed by Kaufmann and colleagues (Fig 1C) [15]. As in the Kaufmann study, the image of Einstein was used in this method.

### 2.3 Classifier

Feature selection for classification uses stepwise linear discriminant analysis (SWLDA), a classification algorithm that selects a set of signal features using ordinary least-squares regression [23]. It iteratively adds significant features and removes the least significant features until either the target number of features is met or a state is reached where no features are added or removed [10]. A score, *y*_*t*_, for a stimulus response is then determined by taking the dot product of the feature vector with the associated EEG signal. Using the score means and variances for target (*μ*_*a*_ and $\sigma_{a}^{2}$) and non-target (*μ*_*n*_ and $\sigma_{n}^{2}$) signals, the likelihood of a signal given a target character, *x*_*t*_, can be determined [21]:

$$p(y_{t}|x_{t})\propto \prod_{i}f(y_{t}^{i}|{x_{t}}^{\left(L\right)})$$

where

$$f(y_{t}^{i}|x_{t})=\left\{ \begin{array}{cc} \frac{1}{\sqrt{2\pi \sigma_{a}^{2}}}e^{-\frac{1}{2\sigma_{a}^{2}}{\left(y_{t}^{i}-\mu_{a}\right)}^{2}} & if\;x_{t}\;contains\;target\\ \frac{1}{\sqrt{2\pi \sigma_{n}^{2}}}e^{-\frac{1}{2\sigma_{n}^{2}}{\left(y_{t}^{i}-\mu_{n}\right)}^{2}} & if\;x_{t}\;does\;not\;contain\;target \end{array}\right.$$

The PF method combines these likelihood probabilities with prior knowledge about language structure to decide the optimal character given the observed signal by estimating the probability distribution over possible outputs [26]. This distribution is created by sampling a batch of possible realizations of the model called particles, which move through states in the language model independently, based on transition probabilities. After each character selection, particles are resampled based on weights derived from observed EEG responses, effectively removing low probability realizations and replacing them with more likely realizations. The algorithm then estimates a probability distribution over the possible output strings by computing a histogram of the particles after they have moved through the model.

When a user begins using the system, a set of *P* particles is generated with an empty history and a weight equal to 1/*P*. At the start of a new character *t*, a sample *x*_0:*t*−1_ is drawn for each particle, *j*, from the proposal distribution defined by the language model’s transition probabilities from the particle’s history, *x*_0:*t*−1_^(*j*)^.

$${x_{0:t}}^{\left(j\right)}∼p(x_{0:t}|{x_{0:t-1}}^{\left(j\right)})$$

Where *p*(*x*_0:*t*_|*x*_0:*t*−1_) is defined from the language model by finding the frequency of occurrence of substrings in an underlying corpus:

$$p(x_{0:t}|x_{0:t-1})=\frac{c(x_{0},\ldots ,x_{t-1},x_{t})}{c(x_{0},\ldots ,x_{t-1})}$$

where *c*(*x*_0_,…,*x*_*t*−1_,*x*_*t*_) refers to the number of times a word occurs in the corpus that begins with the string ′*x*_0_,…,*x*_*t*−1_,*x*_*t*_′. When a particle transitions between states, its history, *x*_0:*t*_^(*j*)^, is stored to represent the output character sequence associated with that particle. After each stimulus response, the probability weight is computed for each of the particles

$${w_{t}}^{\left(j\right)}\propto p(y_{t}|{x_{t}}^{\left(j\right)})\propto \prod_{i}f(y_{t}^{i}|{x_{t}}^{\left(j\right)})$$

The weights are then normalized and the probability of the current character is found by summing the weights of all particles that end in that character.

$$p(x_{0:t}|y_{1:t})=\sum_{k}{w_{t}}^{\left(k\right)}\delta_{x_{t}}^{{x_{t}}^{\left(k\right)}}$$

where δ is the Kronecker delta. A new batch of particles, ***x***_t_^*^, are then sampled from the current particles, ***x***_*t*_, based on the weight distribution, ***w***_*t*_. Each of the new particles are then assigned an equal weight *w*_t_^*(*j*)^ = 1/*P*. The subject then moves on to the next character and the process then repeats with the new batch of particles.

Dynamic classification was implemented by setting a threshold probability to determine when a decision should be made. The program flashed characters until either the probability of at least one character reaches the threshold or the number of flashes reached the maximum (120). The classifier then selected the character that satisfied has the highest probability. In offline analysis, the speeds, accuracies, and CCPMs were found for threshold probability values between 0 and 1 in increments of 0.01 and the threshold that maximized CCPM was chosen for each subject. This optimization was impractical for online experiments, so a previously reported value of 0.95 was used for all trials [24].

### 2.4 Evaluation

Evaluation of a BCI system must take into account two factors: the ability of the system to achieve the desired result and the amount of time required to reach that result. The efficacy of the system can be measured as the selection accuracy, which we defined as the percentage of characters in the final output that matched the target string. The speed of the system was measured using the selection rate (SR), the inverse of the average time required to make a selection.

As there is a tradeoff between speed and accuracy, a metric is needed which takes both into account. Traditionally, BCI systems use information transfer rate (ITR), which calculates the amount of information conveyed in a system’s output, taking into account the accuracy and the number of possible selections [28]. However, this metric makes several assumptions that are not valid in a natural language communication system, including lack of memory between selections, uniform probability of selection across all characters, and a uniform distribution of errors [29,30]. We include ITR here for context across existing P300 speller results, but focus instead on a simpler metric consisting of the number of correctly selected characters per minute (CCPM), discarding incorrect selections. Significance for all values was tested using Wilcoxon signed-rank tests.

## 3 Results

### 3.1 Offline performance

**Fig 2:**
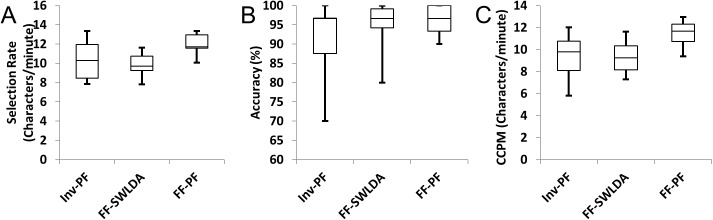
Box plots of the optimal selection rates, accuracies, and correct characters per minute (CCPM) for offline trials using the inverted (Inv) and famous faces (FF) flashing paradigms with either the SWLDA or particle filtering (PF) classifiers with dynamic stopping.

In the preliminary experiment comparing traditional and inverted stimuli, subjects achieved significantly higher typing speeds (10.68 characters/minute versus 9.48 characters/minute) with comparable accuracy (93.39% versus 92.13%) when using inverted stimuli. The main experiment therefore compared performance using inverted and famous faces stimuli. In offline analysis without feedback, two classifiers were used: the standard SWLDA method and the PF method, both with dynamic stopping (Table 1, Fig 2). Using the combination of famous faces and particle filtering classification, there was an average selection rate of 11.97 characters per minute across all subjects, which was significantly higher than those achieved by famous faces with SWLDA (9.78 characters/minute, p = 0.0004) or letter inversion with particle filtering (10.34 characters/minute, p = 0.01). Although the average accuracy achieved by the combination was slightly higher (96.00%) than either of the individual methods (95.00% and 91.67% for famous faces and particle filtering, respectively), accuracy was not significantly different between the three analyses. Overall, the combination of particle filtering yielded an average CCPM of 11.49 characters/min across subjects with all subjects having a value over nine correct characters per minute. This performance was significantly better than that achieved using either famous faces with SWLDA (9.31 chracters/min, p = 0.001) or inverted flashing with particle filtering (9.46 characters/min, p = 0.0003) with nine of the ten subjects having the highest performance using the combined method.

**Table 1:** Optimal selection rates, accuracies, and correct characters per minute (CCPM) for the 10 subjects in offline trials using the inverted (Inv) and famous faces (FF) flashing paradigms with either the SWLDA or particle filtering (PF) classifiers with dynamic stopping.

	SR (selections/min)			ACC (%)			CCPM (characters/min)		
Subject	Inv-PF	FF-SWLDA	FF-PF	Inv-PF	FF-SWLDA	FF-PF	Inv-PF	FF-SWLDA	FF-PF
P	13.36	11.07	12.95	90.00	100.00	100.00	12.02	11.07	12.95
Q	10.64	10.29	11.70	96.67	90.00	100.00	10.29	9.26	11.70
R	12.58	10.88	13.35	86.67	96.67	96.67	10.90	10.51	12.90
S	8.21	9.39	11.57	96.67	96.67	100.00	7.93	9.07	11.57
T	8.30	9.21	11.61	70.00	80.00	90.00	5.81	7.37	10.45
U	12.09	9.57	12.94	96.67	96.67	96.67	11.69	9.26	12.51
V	9.96	11.61	11.75	100.00	100.00	100.00	9.96	11.61	11.75
W	8.91	7.81	10.79	96.67	93.33	93.33	8.61	7.29	10.07
X	11.53	9.81	10.06	83.33	100.00	93.33	9.61	9.81	9.39
Y	7.83	8.13	12.95	100.00	96.67	90.00	7.83	7.86	11.65
Average	10.34	9.78	11.97	91.67	95.00	96.00	9.46	9.31	11.49

### 3.2 Online performance

**Fig 3:**
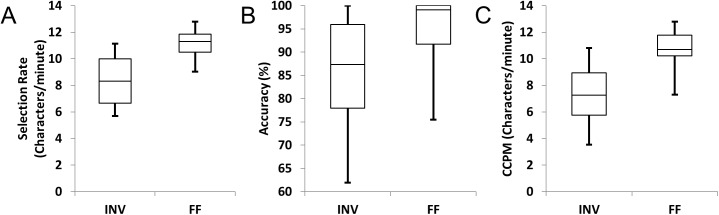
Box plots of the online selection rates, accuracies, and correct characters per minute (CCPM) for each subject using the inverted and famous faces flashing paradigms with the particle filtering classifier.

In online experiments, only the PF classifier was used. All 10 subjects were able to type characters with at least 60% accuracy using each of the flashing paradigms (Table 2, Fig 3). Using the inverting method, nine of the 10 subjects achieved at least 75% accuracy and 6 characters per minute. Using the FF method, all subjects selected characters with at least 75% accuracy, with seven of 10 subjects having accuracies over 98%. All but one of the subjects had typing speeds over 10 characters per minute using the famous faces flashing paradigm.

**Table 2:** Online selection rates, accuracies, and correct characters per minute (CCPM) for each subject using the inverted and famous faces flashing paradigms with the particle filtering classifier.

	SR (selections/min)		ACC (%)		CCPM (characters/min)	
Subject	Inv-PF	FF-PF	Inv-PF	FF-PF	Inv-PF	FF-PF
P	11.02	10.96	98.18	100.00	10.82	10.96
Q	7.36	12.20	75.00	100.00	5.52	12.20
R	9.96	11.90	85.71	100.00	8.54	11.90
S	6.44	11.66	100.00	89.58	6.44	10.44
T	5.70	9.03	61.90	80.77	3.53	7.30
U	10.00	10.45	79.59	100.00	7.96	10.45
V	11.14	12.78	90.38	100.00	10.07	12.78
W	6.27	10.62	77.42	75.47	4.86	8.01
X	9.27	11.63	97.83	98.25	9.07	11.42
Y	7.38	10.34	88.89	98.04	6.56	10.14
Average	8.45	11.16	85.49	94.21	7.33	10.56

All 10 subjects achieved a higher bit rate when using the famous faces flashing paradigm than when using inverting flashes. On average, subjects selected 8.45 characters per minute with 85.49% accuracy, resulting in an average bit rate of 33.86 bits/minute using inverting flashes. When using the famous faces paradigm, subjects achieved significant improvements with an average selection rate of 11.16 characters/minute (32.0% improvement, p = 0.0005), an average accuracy of 94.21% (p = 0.02), CCPM of 10.56 (44.1% improvement, p<0.0001), and an average bit rate of 52.27 bits/minute (54.4% improvement, p = 0.0001).

## 4 Discussion

While there are many active areas of research in improving the P300 speller, relatively little work has been done to combine these improvements. Some of these methods could be mutually exclusive, such as the stimulus presentation pattern presented by Jin et al. [4] and the checkerboard paradigm developed by Townsend et al. [31]. Others, however, can be implemented together, which can potentially produce superior results to either method used individually. Developing a viable system for ALS patient communication will require utilizing many of the improvements that have been developed and it is important that we explore how these components will work together in a final product.

Here, we have demonstrated the performance of the P300 speller when implementing famous faces flashing with a language model-based signal classifier. All subjects achieved their best online performance using the combination of famous faces with the PF classifier. In offline experiments, the improvements were largely a result of a reduction of the number of stimuli required to achieve a similar accuracy. When using the particle filter, the addition of famous faces stimuli increased the selection rate from 10.34 characters/minute to 11.97 characters/minute, an equivalent of reducing the number of flashes by 52%, which is in line with previously published reduction of 45% for famous faces without language modeling [15].Using famous face stimuli with a traditional classifier and using standard flashing with the PF classifier achieved similar results, both of which were substantially higher in terms of selection rate than previously published results using standard methods, which were on the order of 6.5 characters/minute [21]. Combining the methods resulted in the best offline performance for all but one subject. The majority of subjects had worse offline performance using standard flashing compared to inverted stimuli, although famous faces stimuli yield superior results to either alternative method.

There was a decrease in online performance compared to offline analysis, with lower average typing speeds and accuracies for each flashing paradigm. In both cases, the difference was mainly a result of increased selection rate as the accuracy did not significantly differ (p = 0.07 and p = 0.25 for inverted and famous faces flashing, respectively). A similar decrease was seen previously when using language model-based classifiers in an online setting [26]. This decrease could have been caused by the optimization of the probability threshold for each subject in the offline trials. Differences could also have been affected by the target sentence chosen by the users in online trials. Because offline analysis was performed on the training data, all subjects had the same target sentence and therefore benefitted from the language model equally. In online trials, subjects were allowed to choose their own text for free spelling. Sentences that contain words that are common in the language model would have higher prior probabilities, resulting in faster speeds as fewer stimulus responses would be needed for the classifier to reach a decision. Conversely, sentences that are not likely in the language model will have a bias against them and will therefore take longer and are more likely to contain errors. In a realistic system, language models can be individually tailored to reflect text that patients are more likely to type, resulting in further improved results.

### 4.1 Limitations and future directions

The current study was conducted only using healthy volunteers and their performance might not accurately reflect the performance of “locked-in” patients due to additional restrictions such as a lack of gaze control. The PF algorithm will likely have a similar effect in classifying signals from the target population as it is simply a means for improving speed and accuracy and does not affect the appearance of the system for the user. Famous faces stimuli have independently been validated in the target population [17], so it is reasonable to expect the combination of the methods to show an improvement for the target population. Nevertheless, this expectation needs to be tested in a study in the patient population to verify that these improvements will translate into a better system for end users.

## 5 Conclusion

Famous faces stimuli and language model based classification have both been previously shown to greatly improve performance of BCI communication systems. Here, we have shown that the improvements achieved by these methods are complementary and that combining them yields superior results to either method implemented individually in terms of typing speed and information transfer rate. This result has been validated in both online and offline experimental settings. We have also demonstrated that famous faces stimuli are superior to inverted stimuli in addition to standard character intensifications.

## References

[1] Farwell LA, Donchin E. Talking off the top of your head: toward a mental prosthesis utilizing event-related brain potentials. Electroencephalogr Clin Neurophysiol. 1988;70: 510–523. 2461285 10.1016/0013-4694(88)90149-6

[2] Sellers EWE, Krusienski DJDJ, McFarland DJ, Vaughan TM, Wolpaw JR. A P300 event-related potential brain-computer interface (BCI): The effects of matrix size and inter stimulus interval on performance. Biol Psychol. 2006;73: 242–252. doi: 10.1016/j.biopsycho.2006.04.007 16860920 10.1016/j.biopsycho.2006.04.007

[3] Townsend G, Shanahan J, Ryan DB, Sellers EW. A general P300 brain–computer interface presentation paradigm based on performance guided constraints. Neurosci Lett. Elsevier; 2012;531: 63–68. doi: 10.1016/j.neulet.2012.08.041 22960261 10.1016/j.neulet.2012.08.041 PMC3646331

[4] Jin J, Horki P, Brunner C, Wang X, Neuper C, Pfurtscheller G. A new P300 stimulus presentation pattern for EEG-based spelling systems. Biomed Tech. 2010;55: 203–210. 10.1515/BMT.2010.029 20569051

[5] Lu J, Speier W, Hu X, Pouratian N. The effects of stimulus timing features on P300 speller performance. Clin Neurophysiol. Elsevier; 2013;124: 306–314. doi: 10.1016/j.clinph.2012.08.002 22939456 10.1016/j.clinph.2012.08.002 PMC3595069

[6] McFarland DJ, Sarnacki WA, Townsend G, Vaughan T, Wolpaw JR. The P300-based brain-computer interface (BCI): Effects of stimulus rate. Clin Neurophysiol. International Federation of Clinical Neurophysiology; 2011;122: 731–737. doi: 10.1016/j.clinph.2010.10.029 21067970 10.1016/j.clinph.2010.10.029 PMC3050994

[7] Kaper M, Meinicke P, Grossekathoefer U, Lingner T, Ritter H. BCI Competition 2003—Data Set IIb: Support Vector Machines for the P300 Speller Paradigm. IEEE Trans Biomed Eng. 2004;50: 1073–1076. 10.1109/TBME.2004.826698 15188881

[8] Xu N, Gao X, Hong B, Miao X, Gao S, Yang F. BCI Competition 2003—Data Set IIb: Enhancing P300 Wave Detection Using ICA-Based Subspace Projections for BCI Applications. IEEE Trans Biomed Eng. 2004;51: 1067–1072. doi: 10.1109/TBME.2004.826699 15188880 10.1109/TBME.2004.826699

[9] Serby H, Yom-Tov E, Inbar GF. An improved P300-based brain-computer interface. IEEE Trans Neural Syst Rehabil Eng. 2005;13: 89–98. doi: 10.1109/TNSRE.2004.841878 15813410 10.1109/TNSRE.2004.841878

[10] Krusienski DJ, Sellers EW, Cabestaing F, Bayoudh S, McFarland DJ, Vaughan TM, et al A comparison of classification techniques for the P300 Speller. J Neural Eng. 2006;3: 299–305. doi: 10.1088/1741-2560/3/4/007 17124334 10.1088/1741-2560/3/4/007

[11] Liu Y, Zhou Z, Hu D. Comparison of stimulus types in visual P300 speller of brain-computer interfaces. Cognitive Informatics (ICCI), 2010 9th IEEE International Conference on. IEEE; 2010. pp. 273–279.

[12] Takano K, Komatsu T, Hata N, Nakajima Y, Kansaku K. Visual stimuli for the P300 brain–computer interface: a comparison of white/gray and green/blue flicker matrices. Clin Neurophysiol. Elsevier; 2009;120: 1562–1566. doi: 10.1016/j.clinph.2009.06.002 19560965 10.1016/j.clinph.2009.06.002

[13] Salvaris M, Sepulveda F. Visual modifications on the P300 speller BCI paradigm. J Neural Eng. IOP Publishing; 2009;6: 46011. 10.1088/1741-2560/6/4/046011 19602731

[14] Li Y, Bahn S, Nam CS, Lee J. Effects of luminosity contrast and stimulus duration on user performance and preference in a P300-based brain–computer interface. Int J Hum Comput Interact. Taylor & Francis; 2014;30: 151–163.

[15] Kaufmann T, Schulz SM, Grünzinger C, Kübler A. Flashing characters with famous faces improves ERP-based brain–computer interface performance. J Neural Eng. IOP Publishing; 2011;8: 56016. 10.1088/1741-2560/8/5/056016 21934188

[16] Eimer M. Event-related brain potentials distinguish processing stages involved in face perception and recognition. Clin Neurophysiol. Elsevier; 2000;111: 694–705. 10727921 10.1016/s1388-2457(99)00285-0

[17] Kaufmann T, Schulz SM, Köblitz A, Renner G, Wessig C, Kübler A. Face stimuli effectively prevent brain–computer interface inefficiency in patients with neurodegenerative disease. Clin Neurophysiol. 2013;124: 893–900. doi: 10.1016/j.clinph.2012.11.006 23246415 10.1016/j.clinph.2012.11.006

[18] Speier W, Arnold C, Pouratian N. Integrating language models into classifiers for BCI communication: a review. J Neural Eng. IOP Publishing; 2016;13: 31002. 10.1088/1741-2560/13/3/031002 PMC5495144 27153565

[19] Jelinek F. Statistical methods for speech recognition. MIT Press; 1998.

[20] Kindermans P-J, Verschore H, Schrauwen B. A Unified Probabilistic Approach to Improve Spelling in an Event-Related Potential-Based Brain–Computer Interface. Biomed Eng IEEE Trans. IEEE; 2013;60: 2696–2705. 10.1109/TBME.2013.2262524 23674419

[21] Speier W, Arnold C, Lu J, Taira RK, Pouratian N. Natural language processing with dynamic classification improves P300 speller accuracy and bit rate. J Neural Eng. 2011;9: 16004. 10.1088/1741-2560/9/1/016004 PMC3360927 22156110

[22] Park J, Kim K-E. A POMDP approach to optimizing P300 speller BCI paradigm. Neural Syst Rehabil Eng IEEE Trans. IEEE; 2012;20: 584–594. 10.1109/TNSRE.2012.2191979 22510955

[23] Speier W, Knall J, Pouratian N. Unsupervised training of brain-computer interface systems using expectation maximization. Neural Engineering (NER), 2013 6th International IEEE/EMBS Conference on. IEEE; 2013. pp. 707–710.

[24] Speier W, Arnold C, Lu J, Deshpande A, Pouratian N. Integrating language information with a hidden markov model to improve communication rate in the P300 speller. IEEE Trans Neural Syst Rehabil Eng. 2014;22: 678–684. doi: 10.1109/TNSRE.2014.2300091 24760927 10.1109/TNSRE.2014.2300091 PMC4205234

[25] Gordon NJ, Salmond DJ, Smith AFM. Novel approach to nonlinear/non-Gaussian Bayesian state estimation. IEE Proceedings F (Radar and Signal Processing). IET; 1993 pp. 107–113.

[26] Speier W, Arnold CW, Deshpande A, Knall J, Pouratian N. Incorporating advanced language models into the P300 speller using particle filtering. J Neural Eng. 2015;12: 46018. 10.1088/1741-2560/12/4/046018 PMC4509796 26061188

[27] Schalk G, McFarland DJ, Hinterberger T, Birbaumer N, Wolpaw JR. BCI2000: a general-purpose brain-computer interface (BCI) system. IEEE Trans Biomed Eng. 2004;51: 1034–1043. doi: 10.1109/TBME.2004.827072 15188875 10.1109/TBME.2004.827072

[28] Pierce JR. An Introduction to Information Theory. Dover; 1980.

[29] Fatourechi M, Mason SG, Birch GE, Ward RK. Is information transfer rate a suitable performance measure for self-paced brain interface systems? Signal Processing and Information Technology, 2006 IEEE International Symposium on. IEEE; 2006. pp. 212–216.

[30] Speier W, Arnold C, Pouratian N. Evaluating True BCI Communication Rate through Mutual Information and Language Models. PLoS One. 2013;8: e78432 doi: 10.1371/journal.pone.0078432 24167623 10.1371/journal.pone.0078432 PMC3805537

[31] Townsend G, LaPallo BK, Boulay CB, Krusienski DJ, Frye GE, Hauser CK, et al A novel P300-based brain-computer interface stimulus presentation paradigm: Moving beyond rows and columns. Clin Neurophysiol. International Federation of Clinical Neurophysiology; 2010;121: 1109–1120. doi: 10.1016/j.clinph.2010.01.030 20347387 10.1016/j.clinph.2010.01.030 PMC2879474

